# Undergraduate nursing students’ experiences of palliative care in the intensive care unit

**DOI:** 10.1186/s12912-023-01406-6

**Published:** 2023-07-31

**Authors:** Pilaiporn Sukcharoen, Jidapa Polruk, Sununta Lukthitikul, Sadakan Eamchunprathip, Raphatphorn Petchsuk

**Affiliations:** 1 Faculty of Nursing, Suratthani Rajabhat University, Suratthani, Thailand; 2 Faculty of Nursing, Rattana Bundit University, Bangkok, Thailand

**Keywords:** Palliative care, Terminal ill patients, Nursing students

## Abstract

**Background:**

The terminal ill patients in the Intensive Care Unit had physical, mental, social, and spiritual suffering. Nursing students must be aware of own feelings to be able to deliver humanistic care and enable patients to live the rest of lives with dignity. The aim of the study was to investigate experiences of providing palliative care in the Intensive Care Unit (ICU).

**Method:**

This study is a qualitative case study research. In-depth interviews were conducted with the key informants. The key informants were nine Thai third-year nursing students were purposively selected.

**Results:**

The experiences of providing palliative care involved two Themes: (1) self-perception while providing care for terminal ill patients and (2) providing care for terminal ill patients with respect in the patients’ dignity.

**Conclusion:**

These results can be applied to create learning activities to promote nursing students’ self-awareness and enable them to provide humanized care for terminal ill patients.

**Supplementary Information:**

The online version contains supplementary material available at 10.1186/s12912-023-01406-6.

## Background

Terminal ill patients in the Intensive Care Unit (ICU) often have incurable, life-limiting illnesses, and frequently become debilitated and die in this setting [1–3]. The sicknesses are such as trauma, acute heart attack, multiple organ failure, and life-limiting illness. Those have an impact on quality of life [1–3]. Terminal ill patient treatments usually require several complex life-sustaining medical equipment which can engender physical, mental, social, and spiritual suffering. They require the care of the interdisciplinary team. Also, they must stay alone in the ICU without families, contributing to sadness and loneliness [4].

In the past, Thai palliative care concept didn’t apply with the terminal ill patients. Only interdisciplinary team took care before their death [4, 5]. Their families still hope patients recover from complex disease that can’t be cured to reduce patients’ pain and suffering hopefully they would be discharged from the ICU to their house [5]. Therefore, a need for specific care has arisen, especially ICU palliative care to ensure human dignity for patients and their families at end-of-life stage and after the patients’ deaths [2–6].

Palliative care is patient and family centered care aimed at relieving suffering, controlling symptoms and improving quality of life in those with life threatening illnesses [7–9]. The care reduces symptoms and distress, and supports people to accept death, and die peacefully [2, 3, 10–13]. Palliative care, together with including life-prolonging interventions, is provided by critical care professionals and forms a comprehensive approach to care for critically ill patients upon admission to the ICU [14]. The senior nurse or staffs can apply palliative care and allow families to stay in the ICU to be close with patients at end of life [10–12]. Thus, the integration of palliative care has increasingly accepted, especially in ICU terminal ill patients care. The palliative care is a necessary approach in the ICU [2, 3, 5, 14].

Nursing students provide care to real-patients that under control from senior nurses and nursing teacher. After nursing students have completed their studies, they are considered to be a part of healthcare professionals. So, nursing students must study nursing theories and apply knowledge in real-life settings [2, 5, 12, 15]. Specifically, the clinical practice in Adult Nursing Practicum 2 focuses on training nursing students on providing humanistic care for critical and terminal ill patients in the ICU. Nursing students must cooperate with professional nurses in providing care for terminal ill patients in the ICU to meet the standards for nursing education, understand the palliative care concept, and can apply this concept to provide humanistic care both before and after the patients’ deaths [2, 3]. In addition, nursing students are required to have ability to provide palliative care and holistic care to help terminal ill patients and their families to accept the nature of life and death [2, 3, 5] and live the rest of lives with dignity to improve quality of life [2].

However, according to the researcher’s experience of being a supervisor of third-year nursing students in Adult Nursing Practicum 2 focusing on providing humanistic care for terminal ill patients in the ICU, there were concerns that the majority of nursing students had a lack of realization of caring for patients’ emotions, had fear of patients’ suffering and death [3, 16, 17], and had confusion while providing care [2, 3, 5].

Previous studies reported that nursing students mainly provided physical care based on their given responsibilities because they lacked of experience of providing care for dying patients [16, 18–20], including nursing students have a fear of patient death, discouraged, distressed, and have low confidence in care to terminal patients [2, 4, 5], and may connect to lack sympathy with patients and their families [2, 16, 17]. Thus, nursing instructors should offer learning activities that enable nursing students to understand their emotions in the ICU and develop abilities to holistically treat terminal ill patients to let these patients have peace at the end of life [3, 5].

Moreover, previous studies that focused on experiences of palliative care in other countries related to nurses experience the need to exercise autonomy in the ICU on a daily basis [4]. The first-year nursing students experience that the death is more frightening than caring patient in terminal stage [16]. A qualitative study of nursing students’ assessment of the palliative care learning revealed that this concept helped nursing students to know how to communicate with terminal ill patients, and reflected person on death [18], including nursing students providing care for terminal ill patients primarily focused on the promotion of the students’ spirituality in palliative care [2, 3]. A study on experiences of providing care for terminal ill patients in the ICU has not been found, specifically the study with Thai third-year nursing students [17, 21].

Thus, the researcher is interested in investigating experiences of providing palliative care for terminal ill patients in the ICU among Thai nursing students. That is to obtain information on nursing students’ experiences as well as to obtain knowledge about developing learning activities concerning the provision of care for terminal ill patients in the ICU.

## Methods

### Study design

This study utilized a qualitative case study approach to explore the undergraduate nursing students’ experiences of palliative care in the ICU [22]. It was reported according to the relevant guidelines and regulations by including a statement in the methods section is not a method.

### Participants and setting

This study conducted during September to November 2021. This study was undertaken with nine experienced nursing students. The study participant included third- year nursing students from the southern Thailand. Nine Thai third-year nursing students were purposively selected based on these following criteria: (1) third-year students enrolling in Adult Nursing Practicum 2 and (2) had prior 8 weeks of experiences of providing palliative care for terminal ill patients in the ICU. Data about palliative care experiences were collected through in-depth interviews and voice recording by the researcher.

### Research instruments

The research instrument uses semi-structured interview questions. They were developed based on the literature related with the terminal ill patients, palliative care, nursing students, and other related researches [3, 18, 21]. The searching specific key words in Google Scholar and PubMed, including “terminal ill patients”, “the palliative care term”, and “nursing students”. The validation of the information was commenced by three Thai palliative care experts who had experiences in palliative care more than five years. It was edited based on their comments before interviewing. Sample questions: “How were your experiences of providing palliative care for terminal ill patients in the ICU?”, “When providing care for terminal ill patients in the ICU?”, “How did you feel about yourself and terminal ill patients?” and “How did you treat terminal ill patients in the ICU?”

### Data collection

An in-depth semi-structured interview was conducted with each nursing student by the palliative care expertise who has been teaching and done research in palliative care. Each individual was asked to give consent to record the interview and had taken part in the in-depth interviews once or twice, with each time lasting from interview lasted 45–60 min until doesn’t find new information of emotion and feeling of nursing students while taking care terminal ill patients that ensures to the saturation of data. Once data reached saturation, the interview was discontinued. Finally, all time of this qualitative case study research was 12 weeks.

### Data analysis

This study used a qualitative case study design [22]. The researcher transcribed the voice recording word for word. The method employed to generate themes and categories. Thematic analysis was then conducted, applying an inductive method of latent content analysis [23]. The data were interpreted, summarized, and validated. The results of this study were then summarized. Thus, the analyses of the study results were accurate and clearly explained the experiences of providing care for terminal ill patients in the ICU. For validating the results, a triangulation method was conducted, three researchers with expertise in qualitative research reviewed the results. Meanwhile, the results were sent to the key informants to review as well.

## Results

In this study, the interviewees were nine Thai third-year female nursing who had experiences of palliative care in the Intensive Care Unit. (Table 1).

**Table 1:** The participants’ demographic characteristics

Participant’s code	Age (year)	Sex	Palliative Care Experience (weeks)	Marital Status	Level of Education	Position
1	21	female	8	Single	Undergraduate	Nursing student
2	22	female	9	Single	Undergraduate	Nursing student
3	21	female	8	Single	Undergraduate	Nursing student
4	22	female	8	Single	Undergraduate	Nursing student
5	21	female	9	Single	Undergraduate	Nursing student
6	21	female	8	Single	Undergraduate	Nursing student
7	21	female	8	Single	Undergraduate	Nursing student
8	22	female	9	Single	Undergraduate	Nursing student
9	21	female	8	Single	Undergraduate	Nursing student

The analysis revealed two major themes: (1) Self-perception while providing care for terminal ill patients and (2) Providing care for terminal ill patients with respect in the patients’ dignity. (Table 2)

Table 2: The main themes and sub-themes that emerged from the data collected from nursing students’ experiences of palliative care in the Intensive Care Unit.

**Table 2:** Themes and subthemes for undergraduate nursing students’ experiences of palliative care in the Intensive Care Unit

Themes	Subthemes
**Self-perception while providing care for terminal ill patients**	Have self-esteem and realize that they are valuable for patients
	Sympathize with and feel sorry for patients about their suffering
	Accept the death and feel glad when patients are released from suffering
	Have self-control and do not share feelings with patients and families
**Providing care for terminal ill patients with respect in the patients’ dignity**	Providing culturally sensitive holistic humanized care
	Treating patients as if they are family members and provide opportunities for final goodbyes for the families
	Protecting terminal ill patients’ rights and telling them the truth
	Having good relationships and encouraging patients and their families

### Self-perception while providing care for terminal ill patients

It is essential to understand and recognize one’s feelings while providing care for terminal ill patients because these processes promote positive perception about self and care provision. Those who have self-esteem and realize that they are valuable for patients would sympathize with the patients, understand the patients’ suffering, and accept the patients’ deaths. Also, they would be able to regulate their feelings, not to share the feelings with the patients and families.

#### Have self-esteem and realize that they are valuable for patients

Feeling proud of oneself, which was resulted from having an opportunity to help a terminal ill patient, allowed individuals to realize that they were valuable for the patients, have positive attitudes towards oneself and nursing, and feel delightful when providing care to alleviate the patients’ suffering. These feelings resulted in providing humanistic care for patients.

*“Since we’ve provided care, we gave suction, gave bed bath; (we) felt that grandpa had less dyspnea. The patient’s conditions were a lot better. Also, we got a chance to comfort the patient, comfort the families, we got close to the families. Taking care of them makes us feel worthy”* (Participant No. 1).

*“Every time we gave treatment, we talked to grandpa. That formed a bond with the patient. Like, when we gave bed bath, we always initiated a conversation regardless of his response. But, when he made a response, we felt happy and proud that he got better after receiving our care. When telling grandpa to raise his hand and he could make it, that time, we were happy”* (Participant No. 3).

#### Sympathize with and feel sorry for patients about their suffering

When individuals sympathized with and felt sorry for terminal ill patients about their suffering, individuals would understand the patients’ context in which the patients had to endure pain and suffering caused by incurable diseases. Also, they would have desire to provide holistic care for patients, involving physical, mental, social and spiritual aspects to lessen the patients’ suffering.

*“First time I saw, a lot of equipment such as NG tube or other devices attached with the patient’s body. It seemed like the patient’s state was not alert. We were a bit scare. It was the case that we’d never treated previously. But, on second thought, felt sorry for the patient, would like to take care of the patient”* (Participant No. 5).

*“That time, (I) took care of a CA lung case. That time, the patient was on C-PAP. It’s…, the patient was in a lot of pain, the patient’s face was pressed by the device. Look like the patient was in a lot of pain, look pitiful. The patient must have been in pain and did not like it”* (Participant No. 8).

#### Accept the death and feel glad when patients are released from suffering

When individuals accepted patients’ suffering, specifically during the patients’ dying stages and after their deaths; individuals would understand the nature of life and death better. Such understanding linked to feeling of gladness and happiness when the patients were released from their suffering.

*“When the patient was still alive, the patient was gasping, painful. In my mind, (I) thought whether the patient could make it or not. But, for now, that the patient left in peace, (I) noticed that the patient was no longer exhausted. In my mind, (I) felt that (I) don’t want the patient to die. But, on second thought, leaving in peace would be the best way for the patient and it’s better than living in pain and suffering”* (Participant No. 1).

*“When grandma was still intubated, there’s still hope that she could get better. But, look the other ways, grandma left (died), that made her families sad, we were sad too. But, for grandma, that might be good for her because she didn’t have to be in pain any more. Because, when the conditions get worst, getting recover might be hard. If grandma left, (she) might have less suffering”* (Participant No. 7).

#### Have self-control and do not share feelings with patients and families

Being able to regulate one’s emotions while providing care for terminal ill patients was important. Individuals must not share feelings of sadness or distress with terminal ill patients and their families to allow themselves to be able to comfort, encourage, and support the patients and families.

*“We may be unable to tell them apart, between experience and emotions. Like, about emotions, we might cry when a patient is dying, might cry, eyes become moist, sad. It is not supposed to cry like this. (We) should be their rock”* (Participant No. 2).

*“Seemed like the patient breathed faster, the respiratory rate was high. But, after administering medication, (the patient) did not calm. That, (we) felt sad and sorry, like their families. But, as a nurse, (we) must be a rock for their families, and let them see that we were stable. Did not allow (ourselves) to be filled with sad emotions of the patients and their families”* (Participant No. 9).

### Providing care for terminal ill patients with respect in the patients’ dignity

Providing care for terminal ill patients with respect in the patients’ dignity is important. The patients receive holistic care by taking care not only physical, mental, social, but also spiritual aspects. Also, the care responsive to cultures and beliefs. Eventually, the patients and their families have less suffering and spend the rest of their lives peacefully.

#### Providing culturally sensitive holistic humanized care

It was essential to provide humanized care responsive to cultures and beliefs of terminal ill patients and their families. Such care allowed the patients to receive holistic care, incorporating physical, mental, social, and spiritual aspects as well as to spend the rest of their lives in a meaningful way.

*“The case was grandma with CA lung. She was a Buddhist, honored Buddhadasa Bhikkhu. So, we read Dhamma book to her. Reminded her of the Buddha, asked her to chant together and grandma did as we asked. Grandma could not live without medication. Anyway, at that moment, we thought we would do our best”* (Participant No. 6).

*“We must emphasize patient-centered care, provide care that touches patient’s heart, understand their social context and beliefs. Like, being able to provide care for the patients that include all aspects. Focus on providing holistic care, on emotions. One more thing, we must provide emotional support to the patients and their families to let them have peace in mind at the final period”* (Participant No. 8).

#### Treating patients as if they are family members and provide opportunities for final goodbyes for the families

To place importance on providing care for terminal ill patients; especially, providing care as if the patients were family members as well as providing opportunities for final goodbyes for the families enable the patients and their families to have better quality of life and spend time together during the terminal period with happiness.

*“We felt a bond with the patient. My heart wanted to take care of him all the time. When taking temperature, grandpa had a fever, high temperature. When taking his temperature, (we) felt sorry for him, we always tried to give tepid sponge. (We) want him to feel comfortable, want to decrease the temperature. Like, we were bonded with the patient. Like, we were one of the family members”* (Participant No. 4).

*“At that time, the families were afraid to talk to the patient because the patient had drug-resistance infection. When the families visited the patient, they were afraid to touch the patient. We were standing there at that time. We told the families, ‘You may talk, tell the patient that you came, the patient might feel better. Although he could not response, he may hear it”* (Participant No. 5).

#### Protecting terminal ill patients’ rights and telling them the truth

It was essential to protect the rights of terminal ill patients and their families as well as to tell them the truth about the severity of illnesses. That allowed the patients and families to know the true conditions of the patients’ illnesses and understand the nature of life better.

*“Palliative comes with ethics. We must respect the patients. We have to keep a secret. It’s not the patient, …information from the families, I mean the families didn’t want to tell the patient the truth and want us to keep it secret from the patient. So, we have a conflict in my mind – whether or not we should tell the patient about the patient’s conditions. It’s the patient’s body. But, the patient didn’t know anything about it”* (Participant No. 3).

*“When the families got to know that the patient would not get better. This case, the families accepted the patient’s conditions. And, they asked us. So, we explained them. And they understood better. We said, ‘the families could make a decision. A doctor just gave information. Then, the families made a decision about whether or not they would resuscitate the patient if the patient’s heart stop beating”* (Participant No. 4).

#### Having good relationships and encouraging patients and their families

Having good relationships with terminal ill patients and their families, especially effective communication and verbally encouraging and comforting them were important as it helped them to accept treatment and have better quality of life.

*“We are not sure that the patients could hear it, but we will talk about positive things, comfort them, help them to accept their conditions. Like telling them not to worry. For some families who cannot accept it, cry and whine, we will comfort them and recommend them to take good care of the patient because this is end-of-life stage”* (Participant No. 6).

*“When treating the patients, we encourage them. The right way to cheer up the patients verbally is not about saying that they are about to be cured, but about helping them to accept such severe illness. Touching the patients also works. Encouraging is the best way. Like this case, have families. The families could not accept it. At that time, we talked and encouraged the families, helped them to make peace with the situation”* (Participant No. 9).

## Discussion

Thai nursing students’ experiences of palliative care in the ICU found that self-perception while providing care for terminal ill patients consist of have self-esteem and realize that they are valuable for patients. This referred from some nursing students who had fear and disgusting on terminal ill patients’ symptom in the terminal stage of advance disease. They have low self-confidence and less nursing practice skills while providing care [3, 5, 17]. Thus, nursing students must be aware of their own feelings towards the patients, especially towards the patients’ suffering to be able to deliver humanistic care both before and after the patients’ deaths [3, 18, 20].

The palliative care can experience not only the feeling of sympathy and sorrow for patients about their suffering and death but also the feeling pleasant when they are relieved from suffering. This referred from some nursing students had less experience of palliative care for terminal ill patients and lacked communicate with patients and families [16, 18]. Also, nursing students must accept the patients’ distress, deaths and provide care for the families after the patients’ deaths to alleviate pain and suffering of terminal ill patients and families [2, 16, 17].

The nursing students should be able to have self-control, regulate their own feelings, and not share the feelings with the patients’ families, due to the fact nursing students may feel sad, unhappy, and cry with families that connect to loss of holistic care [2, 18, 19]. Thus, nursing students are required to realize their own feelings while providing care for the patients and their families to be able to deliver holistic care for terminal ill patients and enable these patients to live the rest of lives with dignity [3, 5, 12].

In addition, the fact that terminal ill patients in the ICU are incurable and life-limiting illness [1–3]. The patients have to endure suffering [4, 11, 13]. Some cases might show the signs of denying death and loss, although they might not be able to communicate [2, 3] together with the fact that terminal ill patients suffer disease severity and lose ability to communicate, caregivers might not know how the patients would like to be treated and might be unable to holistically satisfy the patients’ needs [4, 11, 13, 20]. Therefore, nursing students providing care for terminal ill patients in the ICU play a key role in reducing the patients’ pain and distress [18–20].

A study on nursing students’ experiences with palliative care revealed that delivering culturally sensitive, holistic, and humanized care, treating patients as if they were family members, can create opportunities for families to have meaningful final goodbyes. This was referred from nursing students must care to centered care, control symptoms and improve quality of life for terminal ill patients [7–9], enable them to accept death and eventually pass away peacefully [3, 10, 11, 13]. Also, nursing students play an important role for terminal ill patients and families; specifically, holistic palliative care and patient-centered care responsive to cultures and beliefs of the patients and their families [2, 3].

Moreover, protecting terminal ill patients’ rights and telling them the truth, and having good relationships and encouraging patients and their families referred to nursing students must the provide care as if the patients are family members, to promote positive relationship with the patients, and to enhance the patients’ quality of life due to the fact terminal ill patients lose ability to take care of themselves and must rely on others [17, 21, 24]. Thus, nursing students must respect their rights and dignity [2, 3, 19, 24]. That is to clarify them about the severity and progression of the diseases at terminal stages. However, before telling the truth, nursing students should assess terminal ill patients and families to prepare to accept the progress of diseases. Those must practice under the control of senior nurses and nursing instructor to ensure in the right direction [2].

The nursing students could provide dramatically help in a specific role in the ICU. It could be such as having interacted and communicate with terminal ill patients and families, spending more time with patients and families, allowing patients and families to share their life stories, and opening opportunity for patients and families to stay in the ICU more than the normal visitation [3, 6]. Moreover, it is the aim of nursing student to facilitate the patients and families to accept the death and spend the final moment together with happiness. They are necessary care person for the provision of palliative care for terminal ill patients in the ICU [3].

In addition, nursing students must provide the holistic care which enable the patients and families to alleviate the suffering [2–[3, 11]–12], accept death, and pass away peacefully [10–13] as well as consider providing care that relevant with Thai tradition and culture such as religion. The patients are dying in peaceful mind after upon the death [2, 3]. Thus, nursing instructors should apply the study results to improve learning model and communication model for coming nursing students to understand how to provide holistic care in the ICU and accept with the patients’ belief, religion, and Thai cultural [3, 6].

## Conclusion

The terminal ill patients in the Intensive Care Unit (ICU) are incurable. They had physical, mental, social, and spiritual suffering. Nursing students must have self-perception while providing care for terminal ill patients, and must be aware of their own feelings towards the patients to be able to deliver humanistic care both before and after the patients’ deaths. In addition, nursing students must respect in the patients’ dignity and apply knowledge in real-life settings for providing humanistic care in the ICU to alleviate the patients’ suffering, holistically fulfill the patients’ last wishes, and eventually pass away peacefully. Therefore, nursing instructors should apply nursing students’ experiences of palliative care to improve and develop nursing education and activities to provide the better care knowledge and experience for terminal ill patients in the ICU.

### Limitations of the study

This study used data obtained only from Thai third-year nursing students who had experiences of palliative care for terminal ill patients in the ICU, including the sample size of the population is quite small. So, it is possible that these results cannot be generalised to other nursing students among other levels of education and other wards.

#### Recommendation

The study results can be applied in the promotion of nursing students’ knowledge and understanding about terminal care provision. Nursing instructors can apply the study results for the improvement of an instructional model such as the communication skills with terminal ill patients and their families, the holistic care skill, and the pain assessment, specifically the provision of learning activities concerning clinical practice for nursing students when providing care for terminal ill patients in the ICU to understand their own feelings while providing care and be able to provide humanized care to the patients. Moreover, another researcher can take this research results to develop by using different instruments to assess skills and attitudes of nursing students while providing care terminal ill patients and families in the ICU.

## Electronic supplementary material

Below is the link to the electronic supplementary material.

Supplementary Material 1

Supplementary Material 2

## Data Availability

The datasets used and/or analyzed during the current study are available from the corresponding author upon reasonable request. All requests relating to data should be addressed to pilaiporn.navynurse@gmail.com.
