# Supplementary material for: Undergraduate nursing students’ experiences of palliative care in the intensive care unit
Source: BMC Nurs. 2023 Jul 31;22:251. doi: 10.1186/s12912-023-01406-6 (PMC10388492; doi:10.1186/s12912-023-01406-6)
Supplement: Supplementary file 1 — Supplementary Material 1 [file 12912_2023_1406_MOESM1_ESM.docx]

| **Quotation** | **coding** | **categories** |
| --- | --- | --- |
| Every time we gave treatment, we talked to grandpa. That formed a bond with the patient. Like, when we gave bed bath, we always initiated a conversation regardless of his response. But, when he made a response, we felt happy and proud that he got better after receiving our care. | - happy - proud | - Self-perception |
| First time I saw, a lot of equipment such as NG tube or other devices attached with the patient’s body. We were a bit scare. It was the case that we’d never treated previously. But, on second thought, felt sorry for the patient, would like to take care of the patient. | - take care of the patient | - Sympathize with patients |
| The patient was gasping, painful. In my mind, (I) thought whether the patient could make it or not. But, for now, that the patient left in peace, (I) noticed that the patient was no longer exhausted. In my mind, (I) felt that (I) don’t want the patient to die. But, on second thought, leaving in peace would be the best way for the patient and it’s better than living in pain and suffering. | - gasping - painful - longer exhausted - pain and suffering. | - feel glad when patients are released from suffering |
| We may be unable to tell them apart, between experience and emotions. Like, about emotions, we might cry when a patient is dying, might cry, eyes become moist, sad. It is not supposed to cry like this. (We) should be their rock. | - we might cry - eyes become moist | - Have self-control |
| Seemed like the patient breathed faster, the respiratory rate was high. But, after administering medication, (the patient) did not calm. That, (we) felt sad and sorry, like their families. But, as a nurse, (we) must be a rock for their families, and let them see that we were stable. | - patient breathed faster | - culturally sensitive |
| The case was grandma with CA lung. She was a Buddhist, honored Buddhadasa Bhikkhu. So, we read Dhamma book to her. Reminded her of the Buddha, asked her to chant together and grandma did as we asked. Grandma could not live without medication. Anyway, at that moment, we thought we would do our best. | - Buddhist - Dhamma - medication | - Treating patients |
| We must emphasize patient-centered care, provide care that touches patient’s heart, understand their social context and beliefs. Like, being able to provide care for the patients that include all aspects. Focus on providing holistic care, on emotions. | - patient-centered care - touches patient’s heart | - Protecting terminal patients’ |
| When taking temperature, grandpa had a fever, high temperature. When taking his temperature, (we) felt sorry for him, we always tried to give tepid sponge. (We) want him to feel comfortable, want to decrease the temperature. Like, we were bonded with the patient. Like, we were one of the family members. | - family members | - Having good relationships |
| Palliative comes with ethics. We must respect the patients. We have to keep a secret. It’s not the patient, …information from the families, I mean the families didn’t want to tell the patient the truth and want us to keep it secret from the patient. So, we have a conflict in my mind – whether or not we should tell the patient about the patient’s conditions. | - tell the patient the truth - keep it secret from the patient | - Have self-esteem |
| We are not sure that the patients could hear it, but we will talk about positive things, comfort them, help them to accept their conditions. For some families who cannot accept it, cry and whine, we will comfort them and recommend them to take good care of the patient because this is end-of-life stage. | - Talk about positive things, | Sympathize with patients |
