# Supplementary material for: Undergraduate nursing students’ experiences of palliative care in the intensive care unit
Source: BMC Nurs. 2023 Jul 31;22:251. doi: 10.1186/s12912-023-01406-6 (PMC10388492; doi:10.1186/s12912-023-01406-6)
Supplement: Supplementary file 2 — Supplementary Material 2 [file 12912_2023_1406_MOESM2_ESM.docx]

**Research instruments**

The semi-structured interview questions

| **Main Question** | **Submain question** |
| --- | --- |
| 1. How were your experiences of providing palliative care for terminal ill patients in the ICU? | - The experiences of the palliative care in the ICU (weeks)? - Your experiences in the palliative care in the ICU? - The ease and difficulty of the palliative care in the ICU? - Like / Dislike on palliative care in the ICU and Why? |
| 1. When providing care for terminal ill patients in the ICU? | - The kind of terminal patient that you take care in the ICU? - When did you start to take care terminal patient in the ICU? - When did you finish to take care terminal patient in the ICU? |
| 1. How did you feel about yourself and the patients? | - Good feeling about yourself while take care terminal patients in the ICU, such as happy, and self-confident and Why? - Bad feeling about yourself while take care terminal patients in the ICU, such as terrible, fear, and unhappy and Why? - Your feeling to terminal patient while you take care terminal patients in the ICU? - Your attitude to terminal patient while you take care terminal patients in the ICU? |
| 1. How did you treat terminal ill patients in the ICU?” | - Nursing practice and method to take care terminal patients and families in the ICU? |
| 1. How did you communicate with terminal ill patients and families in the ICU?” | - Nursing communicate and interact to terminal ill patients and families in the ICU? |
